# The Important Phases of the Root-Canal Problem

**Published:** 1920-04

**Authors:** Julio Endelman


### THE IMPORTANT PHASES OF THE ROOT-CANAL PROBLEM

BY JULIO ENDELMAN, D.D.S.

The pathologic reactions in the tissues which eventually become involved in the infectious process which originates in a carious cavity and which by a process of continuity spread to tissues of the pulp and then to those of the peridental membrane, should be clearly understood by all those who assume the duties of diagnostician in the field of diseases of the pulp and their sequelae. Without any such understanding, without a clear conception of the intimate pathologic phenomena in the periapical tissues and surrounding osseous structures, any attempt at diagnosis would assume the proportions of a humorous parody, if it were not for the far-reaching seriousness of that type of misdirected activity. And right here, in a spirit of kindly criticism, we wish to direct attention at that which impresses us as a most lamentable condition of affairs, though in the midst of well-meaning intentions. The postgraduate course is flourishing and the profession everywhere seems to have awakened from a lethargic state of many decades duration to a realization that the latest information available must become the possession of every earnestly inclined practitioner of his specialty. Courses are eagerly sought on practically every subject of the curriculum except on etiology, pathology and diagnosis.

The very fundamental structure of dentistry is relegated to an oblivion from which the profession, as represented by an overwhelming majority, appears disinclined to institute its rescue. It is, therefore, not in the least surprising that we continue to grope in abject darkness; that we disregard the findings in the field of disease etiology and pathologic histology and content ourselves with a centralization of efforts upon a basis of empiricism and with almost total disregard of the etiologic factors involved. A review of the literature upon the subject-matter under discussion exposes with few exceptions a tendency to undervalue established facts, most of them fundamental—may we not say elementary?—and to discuss the pathology of root involvements upon an individual platform and without any at all or insufficient corroborating facts.

The fundamental pathologic reactions in the pulp, periapical tissues and osseous structure of the alveoli and jaws, while modified by the topography of the parts, differ not from fundamental pathologic reactions in other regions of the body. The pathologic reactions in root-canals of teeth and neighboring structures, thanks to the acceptance of empirical conclusions, has been plunged into an abyss of confusion, absolutely unwarranted by the information everywhere available concerning their nature and significance. The difficulty seems to arise from a policy of indecision and evasiveness with a tendency to compromise upon a ground work of scientific flimsiness. Entirely too much time and energy seem to be allotted to the therapeutic phase of the work. The essential feature of this phase of our problem is the transformation of a septic field into one as free from bacterial life as it is theoretically possible through mechanical and medicinal procedures, regardless of the method employed. We are, or should be, familiar with the efficiency of germicidal agents; the information is available in any standard treatise on materia medica and the strength in which these germicidal agents may be employed with safety to the tissues with which they come into contact is commonplace information. It matters not by what means the bacteria are destroyed and therefore to waste time discussing the relative efficacy of germicides represents a wasteful expenditure of time and leads to the overshadowing of other phases of the problem. It is immaterial whether the agent selected is formo-cresol, formo-creosote, sodium-potassium, chloramine T in aqueous solution, di-chloramine T in an oily chlorinated solution, phenol-sulphonic acid, sulphuric acid in various strengths, or what not. Any one of these various agents when intelligently used will accomplish the desired end; any one of these agents carelessly used will fall short of the mark. The personal equation of the operator, his thoroughness in carrying out an unbroken aseptic chain to the completion of the operation, counts for probably 80 per cent. of the success in root-canal procedures, and the chemical disinfectants and instrumental armamentarium for 20 per cent. The best method is the one which in one's own hands gives the best results.

The question of therapeutics and mechanical manipulations in root-canals is not, in our opinion, the one deserving for the time being of extensive discussion. Disinfection along chemical grounds is the direction in which the best results have been accomplished in the past, and the future will probably witness the advent of new agents which when generated in the nascent state will exhibit the necessary degree of bactericidal power and with it a minimum of tissue injury, thus meeting the requirements of the ideal disinfectants. The phases of root-canal work which, in our opinion, are in need of considerable elucidation are those of etiology, pathology and diagnosis, under pathology including, of course, gross pathology and pathologic histology. Under diagnosis in this connection we are bearing in mind not only a correct differentiation of the existing lesion, but also decisions concerning the advisability of either undertaking the treatment at all of the infected root-canal or of resorting to the extraction of the tooth; and again, if the treatment is undertaken, or ascertaining with some degree of accuracy when the root-canal is ready for its obliteration by filling. These are the phases of the root-canal problem in need of considerable study; these are the phases which should attract the attention of all students of dental pathology.—*Pacific Dental Gazette.*

